# European Correspondence

**Published:** 1859-07

**Authors:** 


### EUROPEAN CORRESPONDENCE.

### ARTICLE VIII.

LONDON, *April* 11*th*, 1859.

*Messrs. Editors.*

"Wee, modest, crimson, tipped flower."

I presume you have deemed me remiss in my promise to correspond for your much esteemed quarterly. Constant journeyings and numerous engagements have prevented me, up to this time, from giving any professional souvenirs of travel. I have also thought it inexpedient to attempt to speak of matters with only a superficial knowledge of persons or things ; and having determined to make a long sojourn in Europe, I still gladly assure you that the forementioned promise shall be faithfully kept ; this brief letter being an earnest of others.

It was my good fortune to be in Scotland during the Centenary of Burns, the now immortal bard. In common with the idle world I participated in the excitement which prevailed, so that with some trouble I visited the parts made peculiarly interesting by the genius of this truly great man of poesy. From his tomb I gathered a beautiful little daisy, endeared to every Scotchman by his feeling pen and to all nature by its simple beauty, and sent it to the best and prettiest of lasses. Most feelingly did I bear witness to the grateful acknowledgment of a nation, when too late to reward its idol. The 25th of January was a day of universal rejoicing in which almost every individual participated, but expressly signalized by public dinners, speeches and toastings. Scotland, so widely celebrated for its love of smoked whiskey and ale, preserved its national decorum wonderfully, for although in its most beautiful city, Edinburgh, which perhaps is the most classically built city of modern Europe, I did not see a single case of intoxication ; the intense enjoyment and patriotism being wholly confined within doors, and characterized by hilarious social pleasure.

The hospitality of this nation, to an individual properly introduced, is something extraordinary, and could the recollections of home ever become obliterated, its fondest attachments would find difficulty in overcoming the hearty welcome, the extreme kindness and the studied comfort everywhere extended to him. This is especially the case in the professional world, and in Edinburgh, the modern Athens in all particulars of classical erudition as well as structural elegance, I received as an American, the most delightful expressions of courtesy. Its School of Medicine is a remarkably fine quadrilateral building, enclosing a large area, from which is found the entrance to the different departments of science and art. Its museum is of course highly creditable, not perhaps what might be expected from the great age of the institution, and its unquestionably superior reputation as the school of instruction. Its library commands general admiration, as much from its magnitude as from its perfect selection and arrangement. In passing I cannot but remark that I feel a great degree of satisfaction in comparing its lecture rooms and operative theatre with those of the Baltimore Dental School, for they strike one as being small, badly lighted, and crushingly heavy.

Prof. Syme is of course the great light of this Institution in connection with Prof. Christison and Prof. Simpson, holding more of a public position than professional, as compared with the two former.

Chloroform is still extensively employed, especially in Edinburgh, and I question much if it will ever cease to be used, since the royal patronage has been extended to this anæsthetic ; still it seem to be quite ignored in this country that this powerful agent comes *after ether*, and that the great probabilities are that it possesses little advantage over it, that though the application of anæsthesia was first made in the United States, more deaths have occurred from its use upon this side than the other. Indeed I fear that from the long contest about its origin, &c., we have lost much of the credit due to American inventive powers, both in material and in instruments of application.

By the civility of Mr. Syme I witnessed his operation of excision of the anterior, two-thirds of the inferior maxilla, and the formation of a new lip. Being placed immediately in front of the table, I had the opportunity of observing closely the cool skill and systematic dexterity of this greatly celebrated surgeon, who gracefully acknowledged the profession's indebtedness, to our side of the water, for many advantages, but especially for the introduction of the metallic ligature which is used in this country almost exclusively. I cannot but suggest here, the propriety of surgeons depending more upon themselves and less upon assistants as leading to a correction of occasional delay, so frequently witnessed, which to the patient, magnifies moments into painful ages.

Whilst enjoying the hospitality of these gentlemen, they expressed their determination of visiting the United States the coming year, where I am sure the whole medical profession will be happy to extend a cordial welcome.

Indeed, it is one continued expression of congratulation, that the medical profession of this country offers the American practitioner, and which I fear is a happy contrast to the estimation borne towards each other in either country, but it will unquestionably induce Americans to return the kind feeling.

Upon every view taken of the medical profession in this country, you are impressed with the unqualified position they hold in all grades of society, forming a great contrast to the imperative and stultifying demarcation ever exercised in this respect towards all other callings, be they what they may, with, perhaps, the single exception of some branches of the law.

A dentist is indeed an icicle, dropped by the whole public, or only accepted as a doubtful expedient in the distant hope of relief from a very indifferent trouble; most generally estimated as producing more injury than benefit. An instance of this state of things was given me by a member of the profession. Mr. Gladstone, M. P., traveling upon one occasion towards the great metropolis, chanced to enter into conversation with his neighbor, whom he found a most intelligent man, and became quite fascinated with the extent and variety of his information, and so expressed himself; he invited the gentleman to his beautiful country residence, and begged the exchange of cards. The traveler stated that he unfortunately had neglected to bring his personal card, and would therefore apologize for presenting a business one which he had by chance about him. Mr. G., upon perceiving that it was a dentist he had been entertained by, immediately gave him the positive cut, taking no further notice of him.

There are, no doubt, some exceptions, but the rule is as stated, the calling is discreditable in the extreme, notwithstanding some are said to be realizing from £20,000 to £25,000 per annum.

Of course, the reason of this is very plain—want of a professional education, for no country in the world will reward and appreciate true ability more generously than England. A student is apprenticed or articled for a term of years, varying from five to seven, during which time he is kept at the bench employed in forming sundry singular, but ingeniously worked pieces for the mouth. He may, if he be fortunate enough to have plenty of money, attend, during or after this period, some course of medical lectures, which would doubtless enable talent to comprehend so far, the science of medicine—he may receive some little personal instruction from a practicing dentist in the operation of extraction, not to any great extent, however, as an immense number of plates are inserted over fangs, so that the skillful excisor of crowns would constitute a chief lesson, followed by various methods of filling with amalgam, which is universally used, and as a consequence most frequently resorted to in the "art of stopping." Gold is sometimes used, but in all candor, shockingly—seldom, and even then, not as is observed in the United States, because it is a material which requires long practice, therefore the general use of the former unfits one for the best use of the latter material.

The result of this is that the British public will not respect members of a calling who have no common source of information or education. Nor can they yield their confidence to such as borrow their claims from a superficial resort to the teaching of another profession, as of course must be ill adapted to the dentist. Indeed, there appears to be such an individual struggle for pre-eminence, such an earnest longing for position, that looking as a stranger upon the scene, we cannot form any definite opinion of the future, for greatness can only come to the profession by a unity of action, and by a common consent to yield individual superiority by imparting such individual power to the mass. This would appear as impossible, as the two societies now formed, are occupying such a position toward each other, but rather striving to individualise themselves, and have not as yet adopted any system which can possibly exalt the profession in its scientific or educational movements. Discussional proceedings usually employ the time of the meetings, and as is most frequently the case, but a small amount of good can be received; as has been stated elsewhere, conviviality has been promoted, but science, we fear, must be in abeyance to the social spirit likely to predominate. Still this is a matter which we earnestly hope will be perfected in the future, and as we are not in the profession, it may not be for our judgment to determine. I am sorry to say that the dentist from the United States is not, only in occasional instances, likely to receive from his profession here so hearty a welcome as he could perhaps wish, and which we feel assured would be extended to any respectable man from this side the water. Indeed, there is a lamentable coolness, an intense indifference in the personalities experienced, and which in some few instances has found vent in the journals, that cannot in many be concealed. Of course this state of things is much to be regretted, especially when viewing the generous accord to English merit, yielded by Americans, and is perhaps a solitary instance in a traveler's experience of John Bull, where he is neither generous or just. But we cannot but say that the great public make ample amends for this by granting a superiority to American dentists, which we fear is not an universal concomitant, but still acknowledged here by the very best and highest of the land, and which we find must compensate for the fault of the profession. The English dentist, as found in his laboratory, deserves great praise for much ingenuity and a very high order of manipulation, and of course excels in this particular the condition of the operative department. Indeed it is in the principles adopted in their practice which prevents the English dentist from becoming the first, practically, in the world, for they are patient, laborious, persistent and long enduring ; a process once adopted, they spend a life time in perfecting it, looking neither to the right or left—their vision is circumscribed by a single object, as instanced in their use of amalgam and bone work—being constitutionally opposed to change, even from bad to better. This may seem to reflect much upon the character or conduct of the two departments, and I cannot but think this reflection would be just, for I have seen mechanical work in the hands of practitioners, as done by their journeymen in the laboratory, the beauty and skill of which I have never seen equalled, but the principle upon which they have been constructed wholly at fault. As to the operative department, I cannot say that I have seen anything worthy of commendation. Indeed, take the English Manual of Dentistry and you are convinced that it is incapable of making a practical man better, or that so far from being calculated to place the profession one step higher in the scale of science, you are rather impressed with the idea that one has only driven a peg to hang his hat on, and has used an ordinary assemblage of words to express what has been promulgated by others. I have recently seen a treatise of this kind, which, upon reading, one cannot but think that its author had been better employed in grinding spar, than in wasting so much ink and paper in teaching the longest road to the poorest end. They are mostly confined to the discussion of matters highly necessary to know, but of themselves wholly impracticable to the dentist, and could be found better expressed in more carefully written treatises. Tracts and pamphlets numerous enough and well executed as to the printing, paper, and mechanical neatness, for they excel the whole world perhaps in this particular.

Their illustrative cuts are also exquisitely done, but alas, too often idle and useless where a correct practice is to be explained or taught. At this moment a little work has been shown to me, in which this is most lamentably exhibited, containing five beautifully executed wood cuts, showing the manner of placing pieces in the mouth to be sustained by the spring so long ago abandoned in the United States—followed by a long description of their use, &c.

This leads me to a striking peculiarity of the profession here, they appear greatly wedded to old practices, and altogether inclined to follow leaders, to do this or that in consequence of some personal precedent much apart from all questions of merit.

Yours, truly, &c.,

